# New symptoms and prevalence of postacute COVID-19 syndrome among nonhospitalized COVID-19 survivors

**DOI:** 10.1038/s41598-022-21289-y

**Published:** 2022-10-08

**Authors:** Asma S. Albtoosh, Ahmad A. Toubasi, Khaled Al Oweidat, Manar M. Hasuneh, Abdullah H. Alshurafa, Daniah L. Alfaqheri, Randa I. Farah

**Affiliations:** 1 Department of Respiratory and Sleep Medicine, Department of Internal Medicine, School of Medicine, The University of Jordan, Amman, Jordan; 2 Faculty of Medicine, The University of Jordan, Amman, 11942 Jordan; 3 Department of Internal Medicine, School of Medicine, The University of Jordan, Amman, Jordan

**Keywords:** Infectious diseases, Microbiology, Medical research

## Abstract

The aim of this study was to assess postacute coronavirus disease 2019 (COVID-19) syndrome (PACS) symptoms according to the onset of the infection while evaluating the effect of COVID-19 vaccination on the symptoms of PACS. We conducted a retrospective single-center cohort study in which nonhospitalized COVID-19 survivors and healthy controls were compared for the occurrence of PACS. The total number of patients in this study was 472. At 6–12 and > 12 months after the infection, COVID-19 survivors had a significantly higher incidence of posttraumatic stress disorder (PTSD) and anxiety than the non-COVID-19 cohort. Furthermore, depression, cognitive deficit, tics, impaired quality of life and general health impairment were significantly more prevalent among COVID-19 survivors at < 6 months, 6–12 months and > 12 months than in the non-COVID-19 cohort. However, respiratory symptoms were significantly more prevalent among COVID-19 survivors only in the first 6 months after infection. In addition, cognitive deficit (OR = 0.15; 95% CI 0.03–0.87) and impaired quality of life (B = − 2.11; 95% CI − 4.21 to − 0.20) were significantly less prevalent among vaccinated COVID-19 survivors than among nonvaccinated survivors. Longitudinal studies are needed to establish the time that should elapse after COVID-19 infection for the symptoms of PACS to appear. Randomized clinical trials are needed to assess the possibility that COVID-19 vaccines might relieve PACS symptoms.

## Introduction

The severe acute respiratory syndrome coronavirus 2 (SARS-CoV-2) pandemic continues to be a major public health burden and is responsible for significant morbidity and mortality, with upward of 260 million cases reported worldwide at present. The symptoms of acute illness range from mild to severe, with a wide range of possible symptoms presenting 2–14 days after exposure^1,2^.

It was reported early in the pandemic that the COVID-19 fatality rate was 1–5%^3^; however, the rapid spread of the virus resulted in deleterious effects on public health and expectations of a potential drop in life expectancy worldwide in different magnitudes depending on the region^4^. The enormous mortality toll of the pandemic resulted in projections of a decline in life expectancy of more than 1 year in the United States^5^. In addition to the mortality toll, the persistence of symptoms beyond four weeks of the initial episode was reported in a large proportion of the affected patients and collectively called postacute COVID-19 syndrome (PACS). This term encompasses a wide range of symptoms that appear after infection and lasts weeks to months, including new or ongoing symptoms, multiorgan effects of COVID-19 and the effects of COVID-19 treatment or hospitalization^6,7^. The most commonly reported manifestations include general, respiratory, cardiovascular, neurological, gastrointestinal, musculoskeletal, psychiatric and dermatological symptoms, among others^8,9^.

Several factors were implicated in increasing the likelihood of developing PACS. Some of these factors included the COVID-19 course, sex and the survivor’s past medical history^10^. A more severe COVID-19 course was associated with the development of subsequent neurological, pulmonary, musculoskeletal, and psychiatric diagnoses and general fatigue^10,11^, whereas milder cases were associated with the development of anosmia and ageusia^10^. The presence of more than five symptoms in the first week of illness was also associated with PACS^9^. Additionally, persistent fatigue, psychological symptoms, and other sequelae were more likely to occur in women^8,9^. Other individuals who had an increased risk of PACS were young/middle-aged patients, patients above the age of 70, and patients with multiple comorbidities^12,13^.

It was estimated that more than 40% of COVID-19 survivors suffer from PACS^13^. In addition, a recent meta-analysis demonstrated that the highest prevalence of PACS was in Asia, with a prevalence of 49%^13^. The studies performed regarding PACS indicated that COVID-19 survivors might suffer from long-term debilitating symptoms after their recovery^12^. Although several studies have characterized these symptoms, most of them were performed on hospitalized COVID-19 patients, and new symptoms are still being reported, such as movement disorders^14^. In addition, the duration of time that should elapse for these symptoms to subside has not yet been studied. Moreover, online polls among patients with PACS showed that patients reported a reduction in their symptoms after receiving the COVID-19 vaccine^15^. Furthermore, several studies reported an increase in tics and tic-like symptoms during the COVID-19 pandemic, yet no studies have investigated their association with COVID-19 infection^16^.

The primary aims of this study were to assess PACS symptoms among nonhospitalized COVID-19 patients, including quality of life, cognitive function, tic symptoms, respiratory symptoms, posttraumatic stress disorder, anxiety and depression, and to evaluate these symptoms according to the time of COVID-19 infection by comparing nonhospitalized COVID-19 survivors with non-COVID-19 controls as well as to report the newly reported symptoms of PACS (tics). The secondary aim was to evaluate the effect of the COVID-19 vaccine on PACS symptoms.

## Methods

### Study design and participants

A retrospective cohort study was conducted at Jordan University Hospital (JUH), a tertiary hospital located in Amman, Jordan. The current population of Jordan is 10,909,567, of whom 53.1% are men. The median age of the Jordanian population is 23.5 years. Regarding comorbidities, approximately 15% of the Jordanian population has diabetes, and 20% of the population has hypertension. The participants were recruited by convenience sampling of patients at JUH. All patients admitted to the hospital ward not due to or during the COVID-19 infection or who visited the outpatient clinics between September 29th and November 11th, 2021, were eligible to participate in this study. Two cohorts were included in the study: (1) all COVID-19 survivors who were not hospitalized due to COVID-19 or during the COVID-19 infection period regardless of the interval between infection and enrollment in this study and (2) all individuals who never tested positive for COVID-19. The ascertainment of COVID-19 infection was based on reverse transcription polymerase chain reaction (RT-PCR) nasopharyngeal swab results. The history of COVID-19 infection was evaluated in multiple steps. First, the patient was asked about a history of a positive RT-PCR swab for COVID-19 and whether the patient underwent the test at our institution or elsewhere. If the patient underwent the test at our institution, the test result was confirmed using medical records. If the patient underwent the test outside our institution, the patient was asked to provide the confirmatory test results sent by the Jordanian Ministry of Health to the patient’s phone. Self-reported results without confirmatory tests were not accepted, and the patient was subsequently excluded from the study. Patients were excluded if they were hospitalized due to or during the COVID-19 infection, did not agree to participate, withdrew their consent anytime during the interview, had missing data regarding infection and vaccination status or had psychiatric or dementia disorders based on the medical records regardless of the severity of the disorder. This study was approved by the Institutional Review Board at JUH (IRB#202123152), and the participants were provided with the aims of the study prior to the initiation of the interview. Written informed consent was obtained from all patients who agreed to participate in the study. This research was conducted in accordance with the Declaration of Helsinki regulations and its relevant guidelines.

### Data collection procedure

The data were collected using structured face-to-face interviews administered by the members of the research team, who were trained to interview patients via online and face-to-face sessions prior to the conduction of the study. The training involved explanation of the interview questions as well as multiple simulations of the interview procedure in the hospital setting. The data collection process was composed of two steps. The first step was to interview the patients who agreed to participate for the occurrence of PACS symptoms and the presence of tics. PACS components were assessed using eight scales, including the EuroQol five-dimension five-level assessment (EQ-5D-5L), EuroQol Visual Analogue Scale (EQ-VAS), Impact of Event Scale (IES-6), Generalized Anxiety Disorder 7-item scale (GAD-7), Patient Health Questionnaire 9 (PHQ-9), Modified Medical Research Council scale (mMRC), Breathlessness, Sputum and Cough Scale (BCSS), and Montreal Cognitive Assessment (MoCA)^17–23^. These scales were shown to comprehensively screen for PACS symptoms among COVID-19 survivors by Johns Hopkins Hospital^24^. Patient characteristics were extracted from the hospital electronic medical system (EMS), including (1) patient demographics (age, sex and body mass index (BMI)), (2) past medical history, (3) actively prescribed medications extracted from the patient medical records profile and patients’ pharmacy refills in the past 3 months, (4) history of COVID-19 infection and date of that infection, (5) COVID-19 vaccination status, and (6) Hospitalization status during the interview which was done after the patient recovery from the COVID-19 infection. It is important to mention that hospitalization status during the COVID-19 infection was not part of the data collection procedure as any patient hospitalized during the COVID-19 infection period was excluded from the study.

### Outcome measures

The primary outcomes were quality of life assessed by the EQ-5D-5L assessment, general health status assessed by the EQ-VAS, cognitive function assessed by the MoCA, and tic symptoms and respiratory symptoms assessed by the BCSS and mMRC scale, respectively. The secondary outcomes were posttraumatic stress disorder (PTSD) assessed by the IES-6, anxiety assessed by the GAD-7 scale, and depression assessed by the PHQ-9. The EQ-5D-5L assessment describes five domains for quality of life, namely, mobility, self-care, usual activities, pain/discomfort, and anxiety/depression. Each of these aspects is scored from 1 to 5, with higher scores indicating higher impairment for each domain. The aforementioned domains were used to calculate the EQ-5D-5L index value, which is a valid index that estimates overall quality of life^25^. The EQ-5D-5L index ranges between 0 and 1, with higher scores indicating lower quality of life^25^. The way of calculating the index value is described in Supplementary Material 1. The EQ-5D-5L assessment also includes a vertical visual analog scale (EQ-VAS) that allows participants to grade their perceived health on the day of the interview on a scale from 0 to 100, with higher scores indicating better subjective health^23^. The MoCA is a validated tool that assesses mild cognitive impairment (MCI) with 92.3% sensitivity and 85.7% specificity. The maximum possible score that can be achieved is 30, with a score below 26 indicating MCI^19^. The tics were assessed among the participants by asking them whether they had experienced repetitive movements. Whenever a patient with a history of COVID-19 infection experienced such movements, the patient was asked whether these movements were experienced prior to or after a positive COVID-19 test. Additionally, the patients were asked about the nature of the experienced movements in addition to a previous history of movement or tic-related disorders. Patients in both cohorts were considered to be positive for tic assessment if they experienced repetitive movements and did not have any history of movement disorder. COVID-19 survivors were considered positive for tic assessment if they met the aforementioned criteria in addition to experiencing these symptoms after a positive test for the COVID-19 survivor cohort. Furthermore, the BCSS is a 3-item instrument that rates breathlessness, cough and sputum on a 5-point Likert scale graded from 0 to 4, where 0 indicates no symptoms and 4 indicates severe symptoms^18^. Additionally, the mMRC scale was used to assess respiratory symptoms. This scale is composed of 5 items and is used to determine the associated disability and impact of breathlessness on everyday life, graded from 0 to 4, with higher scores indicating more severe symptoms^22^. The IES-6 is an instrument used to screen for PTSD that results from any stressful event, including trauma, natural disasters, personal events and chronic comorbidities. A cutoff score of 1.75 has a sensitivity of 88% and a specificity of 85% in identifying PTSD^17^. The GAD-7 scale is a valid instrument used to screen for anxiety, with a cutoff point of 10 or greater having a sensitivity of 89% and specificity of 82%^20^. Finally, the PHQ-9 is an instrument that is used to assess symptoms of depression. Its cutoff score for diagnosing depression of 8 or higher has a sensitivity of 88.2% and a specificity of 86.6%^21^. Moreover, patients who scored > 1.75 on the IES-6, ≥ 10 on the GAD-7 scale and ≥ 8 on the PHQ-9 were considered positive for PTSD, anxiety and depression, respectively. The COVID-19 survivors were stratified into 3 categories according to the duration between infection and interview: < 6 months, 6–12 months and > 12 months to demonstrate the changes in the prevalence of PACS symptoms with time. In addition, the COVID-19 survivors were categorized according to their vaccination status into vaccinated and unvaccinated groups, and the effect of vaccination on PACS symptoms was tested. Patients had to receive the initial series doses to be considered vaccinated (2 doses of Pfizer or Sinopharm vaccine or 1 dose of Johnson & Johnson) regardless of whether they received a booster shot. Vaccination status and the received vaccine types had to be confirmed by the patient through a vaccine certificate provided by the Jordanian Ministry of Health.

### Statistical analysis

Data analysis was performed using IBM SPSS v.25. Demographic characteristics were summarized using percentages for categorical variables and the means with their corresponding standard deviations for continuous variables. Chi-square and t tests were used to assess the differences in the demographic characteristics and outcome measures between the COVID-19 survivors and the non-COVID-19 cohorts. Also, Odds Ratio (OR) and Cohen’s d were used as effect measures to estimate the differences in the categorical and continuous demographic variables, respectively between COVID-19 survivors and non-COVID-19 cohort. Multivariate logistic and linear regression models were used to assess the effect of COVID-19 infection on the outcomes. Furthermore, to identify the pattern of PACS symptom prevalence changes with time, COVID-19 infection durations (< 6 months, 6–12 months and > 12 months) were compared for the presence of PACS symptoms with the non-COVID-19 cohort as a control group (reference) using multivariate logistic and linear regression models. In addition, to examine the effect of vaccination on PACS symptoms, a comparison of the occurrence of PACS symptoms between the vaccinated and unvaccinated COVID-19 survivor groups was performed using multivariate logistic and linear regression models. These models were adjusted for age, sex, BMI, hospitalization status during the interview, comorbidities (diabetes mellitus, respiratory comorbidities, cardiovascular comorbidities, Chronic Kidney Disease (CKD), cancer, autoimmune diseases, Gastrointestinal disorders, neurological diseases, and others) and medication use (antidiabetic drugs, antihypertensives, antiplatelets/anticoagulants, inhalers, dyslipidemia drugs, Proton Pump Inhibitors (PPIs)/H2 blockers and others). All significance tests were two-sided, and a P value of less than 0.05 was considered statistically significant. P-values were not adjusted for multiple comparison as studies showed that although adjustments reduce type I error frequency, it increases the risk of type II error^26^.

## Results

We invited 730 patients to participate in this study, among whom 200 refused to participate, 22 withdrew their consent during the interview and 36 were excluded because they had psychiatric or dementia disorders or because they were missing data regarding infection and vaccination status. The participants flow diagram is described in Supplementary Material 2. The total number of participants who completed the interview was 472, with a response rate of 64.7%. Among those patients, 26.5% were COVID-19 survivors (125/472), while the rest had never tested positive for COVID-19. The majority of the COVID-19 patients were infected > 12 months before the interview (43.2%). However, 28.8% and 28.0% were infected < 6 months and 6–12 months before the time of the assessment, respectively.

### General characteristics

The COVID-19 and non-COVID-19 control groups were comparable in all demographic characteristics except for sex and smoking status (P value = 0.008 and 0.016, respectively). The percentage of men in the non-COVID-19 group (64.0%) was significantly higher than that in the COVID-19 group (50.4%). In addition, the percentage of smokers (42.7%) was significantly higher in the non-COVID-19 group than in the COVID-19 group (30.4%). Furthermore, there were no significant differences between the COVID-19 and non-COVID-19 groups in terms of comorbidities or medication use except for GI disorders, which were more prevalent in the non-COVID-19 group (P value = 0.040), and inhaler use, which was higher in the COVID-19 group (P value = 0.027). Additionally, there was no significant difference between the COVID-19 group and the non-COVID-19 group in the number of patients who were admitted to the hospital ward during the interview. Moreover, the incidence of PTSD, anxiety, depression, cognitive deficit, and tics was significantly higher in the COVID-19 group than in the non-COVID-19 group. The COVID-19 group had a significantly lower mean EQ-VAS score than the non-COVID-19 group (P value = 0.000). On the other hand, the mean EQ-5D-5L assessment, mMRC scale and BCSS scores were significantly higher in the COVID-19 group than in the non-COVID-19 group. The detailed characteristics of the COVID-19 and non-COVID-19 cohorts are described in Table 1.

**Table 1:** The characteristics of COVID-19 survivors and non-COVID-19 cohorts.

Variables	Non-COVID-19 controls (n = 347)	COVID-19 survivors (n = 125)	Effect size (Cohen’s d/OR)	p value
**Sex**				
Men	222 (64.0)	63 (50.4)	0.572	0.008*
Women	125	62		
Age	40.818 ± 14.229	39.270 ± 14.644	0.109	0.301
BMI	23.73 ± 10.16	24.65 ± 17.74	0.064	0.494
Smoking	148 (42.7)	38 (30.4)	0.587	0.016*
Hospitalization status during the interview	183 (52.7)	76 (60.8)	1.390	0.120
**Vaccination status**				
Vaccinated	250 (72.0)	91 (72.8)	1.03	0.872
Unvaccinated	97 (28.0)	34 (27.2)		
**Duration between infection and interview (Intervals)**				
Not infected	347	–	–	–
Less than 6 months	–	36 (28.8)	–	
6–12 months	–	35 (28.0)	–	
More than 12 months	–	54 (43.2)	–	
Diabetes mellitus	76 (21.9)	22 (17.6)	0.762	0.309
Respiratory comorbidities	25 (7.2)	6 (4.8)	0.649	0.484
Cardiovascular comorbidities	83 (23.9)	28 (22.4)	0.918	0.477
CKD	14 (4.0)	5 (4.0)	0.991	0.987
Cancer	29 (8.4)	11 (8.8)	1.058	0.879
Autoimmune diseases	24 (6.9)	8 (6.4)	0.920	0.844
GI disorders	17 (4.9)	1 (0.8)	0.157	0.040*
Neurological diseases	13 (3.7)	2 (1.6)	0.418	0.241
Others	24 (6.9)	8 (6.4)	0.920	0.844
Antidiabetic drugs	28 (8.1)	6 (4.8)	0.574	0.225
Antihypertensives	6 (1.7)	4 (3.2)	1.879	0.328
Antiplatelets/anticoagulants	13 (3.7)	3 (2.4)	0.632	0.476
Inhalers	1 (0.3)	3 (2.4)	8.508	0.027*
Dyslipidemia drugs	5 (1.4)	2 (1.6)	1.112	0.900
PPIs/H2 blockers	3 (0.9)	0 (0.0)	0.392	0.297
Others	14 (4.0)	4 (3.2)	0.786	0.676
PTSD	207 (59.7)	105 (84.0)	3.551	0.000*
Anxiety	67 (19.3)	48 (38.4)	2.605	0.000*
Depression	158 (45.5)	94 (75.2)	3.627	0.000**
Cognitive deficit	166 (47.8)	90 (72.2)	2.804	0.000*
Tics	24 (6.9)	23 (18.4)	3.035	0.000*
General health status (VAS)	73.98 ± 17.95	67.20 ± 19.286	0.364	0.000*
Quality of life (EQ-5D-5L assessment score)	0.39 ± 0.36	0.66 ± 0.32	0.793	0.000*
MMRC scale score	0.65 ± 1.03	0.88 ± 1.229	0.203	0.042*
BCSS score	1.36 ± 1.82	1.76 ± 2.06	0.206	0.042*

This table shows the differences between COVID-19 survivors and non-COVID-19 patients in terms of general characteristics and outcomes.

*P value < 0.05.

*BMI* Body mass index, *COPD* Chronic obstructive pulmonary disease, *OSA* Obstructive sleep apnea, *HTN* Hypertension, *HF* Heart failure, *IHD* Ischemic heart disease, *CKD* Chronic kidney disease, *CVAs* Cerebrovascular diseases, *ACEI* Angiotensin-converting enzyme inhibitor, *ARB* Angiotensin receptor blocker, *CCB* Calcium channel blocker, *PPIs* Proton pump inhibitors, *ACH* Acetyl choline, *PTSD* Posttraumatic stress disorder, *VAS* Visual Analogue Scale, *EQ-5D-5L* EuroQol 5-dimension 5-level assessment, *MMRC* Modified Medical Research Council scale, *BCSS* Breathlessness, Cough, and Sputum Scale.

### COVID-19 survivors and PACS

In the multivariate regression analysis, COVID-19 infection was significantly associated with PTSD, anxiety, depression, cognitive deficit, tics, EQ-VAS score, EQ-5D-5L assessment score, and BCSS score. In contrast, COVID-19 infection was not significantly associated with mMRC scale score. The COVID-19 survivor group had a significantly higher prevalence of PTSD than the non-COVID-19 survivor group (OR = 3.99; 95% CI 2.08–7.64). Similarly, a significantly higher prevalence of anxiety (OR = 2.40; 95% CI 1.41–4.45) and depression (OR = 4.11; 95% CI 2.25–7.48) was demonstrated in the COVID-19 survivor group than in the non-COVID-19 survivor group. Moreover, COVID-19 infection was associated with a higher prevalence of tics (OR = 4.90; 95% CI 2.16–11.12) and cognitive deficit (OR = 3.61; 95% CI 2.00–6.28). In addition, the COVID-19 survivor group had a significantly higher EQ-5D-5L assessment score (B = 1.81; 95% CI 1.10–2.92) and BCSS score (B = 0.56; 95% CI 0.15–0.97) than the non-COVID-19 survivor group. However, COVID-19 infection was significantly associated with a lower EQ-VAS score (B =  − 8.31; 95%; 95% CI − 12.26 to − 4.52) (Table 2).

**Table 2:** Multivariate logistic regression of COVID-19 survivors and PACS.

Response	AOR (95% CI)	*p value*
**PTSD**		
COVID-19 survivors	3.99 (2.08–7.64)	0.000*
Non-COVID-19 patients	Reference	Reference
**Anxiety**		
COVID-19 survivors	2.4 (1.41–4.45)	0.001*
Non-COVID-19 patients	Reference	Reference
**Depression**		
COVID-19 survivors	4.11 (2.25–7.48)	0.000*
Non-COVID-19 patients	Reference	Reference
**Cognitive deficit**		
COVID-19 survivors	3.61 (2.00–6.28)	0.000*
Non-COVID-19 patients	Reference	Reference
**Tics**		
COVID-19 survivors	4.90 (2.16–11.12)	0.000*
Non-COVID-19 patients	Reference	Reference

Response	B (95% CI)	*p value*
**Quality of Life (EQ-5D-5L Assessment)**		
COVID-19 survivors	1.81 (1.10–2.92)	0.000*
Non-COVID-19 patients	Reference	Reference
**General Health Status (EQ-VAS)**		
COVID-19 survivors	− 8.31 (− 12.26 to − 4.52)	0.000*
Non-COVID-19 patients	Reference	Reference
**MMRC Scale**		
COVID-19 survivors	0.12 (− 0.12 to 0.41)	0.218
Non-COVID-19 patients	Reference	Reference
**BCSS**		
COVID-19 survivors	0.56 (0.15–0.97)	0.008*
Non-COVID-19 patients	Reference	Reference

This table shows the differences in PACS occurrence between COVID-19 survivors and non-COVID-19 patients.

*P value < 0.05.

*PTSD* Posttraumatic stress disorder, *VAS* Visual Analogue Scale, *EQ-5D-5L* EuroQol 5-dimension 5-level assessment, *MMRC* Modified Medical Research Council scale, *BCSS* Breathlessness, Cough, and Sputum Scale.

### COVID-19 infection duration and PACS

After stratification of COVID-19 survivors into groups based on the duration between infection and the time of the interview, each stratum of duration between COVID-19 infection and the interview was compared to that in the non-COVID-19 group for the occurrence of the outcome measures. The results showed that the odds of PTSD were significantly higher in the 6–12 month group (OR = 4.02; 95% CI 1.33–12.15) and the > 12 month group (OR = 5.17; 95% CI 1.91–14.00) than in the non-COVID-19 cohort. Similarly, the odds of anxiety were significantly higher in the 6–12 month group (OR = 4.41; 95% CI 1.66–11.64) and the > 12 month group (OR = 2.19; 95% CI 1.04–4.63) than in the non-COVID-19 cohort. However, the odds of both anxiety and PTSD were not significantly different between the < 6 month group and the non-COVID-19 cohort. On the other hand, depression was significantly higher in the < 6 month group (OR = 2.87; 95% CI 1.11–7.41), 6–12 month group (OR = 5.65; 95% CI 1.90–16.87) and > 12 month group (OR = 4.43; 95% CI 1.94–10.15) than in the non-COVID-19 cohort. Comparably, cognitive deficits were significantly higher in the < 6 month group (OR = 3.43; 95% CI 1.30–9.00), 6–12 month group (OR = 5.25; 95% CI 1.88–14.70) and > 12 month group (OR = 2.92; 95% CI 1.37–6.25) than in the non-COVID-19 cohort. Additionally, the odds of developing tics were significantly higher in the < 6 month group (OR = 5.35; 95% CI 1.59–17.75), 6–12 month group (OR = 8.43; 95% CI 2.42–29.32) and > 12 month group (OR = 3.41; 95% CI 1.20–9.72) than in the non-COVID-19 cohort. The mean EQ-5D-5L assessment score was significantly higher in the 6–12 month group (B = 2.09; 95% CI 0.67–3.70) and > 12 month group (B = 1.72; 95% CI 0.64–3.09) than in the non-COVID-19 cohort, while the score was not significantly different between the < 6 month group and the non-COVID-19 cohort. Moreover, the mean EQ-VAS score was significantly higher in the < 6 month group (B =  − 7.24; 95% CI − 13.99 to − 0.50) and > 12 month group (B =  − 11.28; 95% CI − 16.53 to − 6.02) than in the non-COVID-19 cohort, whereas the 6–12 month group was not significantly different in terms of EQ-VAS score compared to the non-COVID-19 cohort. On the other hand, the mean BCSS score was significantly higher in the < 6 month group than in the non-COVID-19 group (B = 1.02; 95% CI 0.32–1.71). However, this difference was not significant in the 6–12 month or the > 12 month groups compared to the non-COVID-19 cohort. There was no significant difference in the mMRC scale scores between any of the infection duration categories and the non-COVID-19 group (Table 3).

**Table 3:** Multivariate logistic regression of the COVID-19 infection durations and PACS.

Response	AOR (95% CI)	*p value*
**PTSD**		
Control	Reference	Reference
Less than 6 months	2.72 (0.93–8.11)	0.075
6–12 months	4.02 (1.33–12.15)	0.014*
More than 12 months	5.17 (1.91–14.00)	0.001*
**Anxiety**		
Control	Reference	Reference
Less than 6 months	1.85 (0.73–4.73)	0.197
6–12 months	4.41 (1.66–11.64)	0.002*
More than 12 months	2.19 (1.04–4.63)	0.040*
**Depression**		
Control	Reference	Reference
Less than 6 months	2.87 (1.11–7.41)	0.029*
6–12 months	5.65 (1.90–16.87)	0.002*
More than 12 months	4.43 (1.94–10.15)	0.000*
**Cognitive deficit**		
Control	Reference	Reference
Less than 6 months	3.43 (1.30–9.00)	0.012*
6–12 months	5.25 (1.88–14.70	0.002*
More than 12 months	2.92 (1.37–6.25)	0.006*
**Tics**		
Control	Reference	Reference
Less than 6 months	5.35 (1.59–17.75)	0.006*
6–12 months	8.43 (2.42–29.32)	0.001*
More than 12 months	3.41 (1.20–9.72)	0.022*

Response	B (95% CI)	*p value*
**Quality of Life (EQ-5D-5L Assessment)**		
Control	Reference	Reference
Less than 6 months	1.21 (− 0.01 to 2.73)	0.051
6–12 months	2.09 (0.67–3.70)	0.004*
More than 12 months	1.72 (0.64–3.09)	0.002*
**General Health Status (EQ-VAS)**		
Control	Reference	Reference
Less than 6 months	− 7.24 (− 13.99 to − 0.50)	0.036*
6–12 months	− 4.81 (− 10.93 to 1.35)	0.125
More than 12 months	− 11.28 (− 16.53 to − 6.02)	0.000*
**MMRC Scale**		
Control	Reference	Reference
Less than 6 months	0.17 (− 0.22 to 0.55)	0.389
6–12 months	0.22 (− 0.16 to 0.60)	0.250
More than 12 months	0.11 (− 0.28 to 0.43)	0.646
**BCSS**		
Control	Reference	Reference
Less than 6 months	1.02 (0.32–1.71)	0.004*
6–12 months	0.59 (− 0.076 to 1.26)	0.082
More than 12 months	0.26 (− 0.28 to 0.80)	0.346

This table shows the comparison between the COVID-19 infection durations of COVID-19 patients and the controls to assess the prevalence of PACS symptoms among COVID-19 survivors.

*P value < 0.05.

*PTSD* Posttraumatic stress disorder, *VAS* Visual Analogue Scale, *EQ-5D-5L* EuroQol 5-dimension 5-level assessment, *MMRC* Modified Medical Research Council scale, *BCSS* Breathlessness, Cough, and Sputum Scale.

### Vaccination status of COVID-19 survivors and PACS

In the comparison according to vaccination status for the odds of developing PACS outcomes in the COVID-19 survivor cohort, only cognitive deficit and EQ-5D-5L assessment scores were significantly different between the vaccinated and nonvaccinated groups. The cognitive deficit prevalence (OR = 0.15; 95% CI 0.03–0.87) and the EQ-5D-5L assessment scores (B =  − 2.11; 95% CI − 4.21 to − 0.20) were significantly lower in the vaccinated group than in the nonvaccinated group (Table 4).

**Table 4:** Multivariate logistic regression of COVID-19 vaccination status and PACS.

Response	AOR (95% CI)	*p value*
**PTSD**		
Vaccinated	0.68 (0.09–4.92)	0.700
Not vaccinated	Reference	Reference
**Anxiety**		
Vaccinated	0.35 (0.10–1.25)	0.107
Not vaccinated	Reference	Reference
**Depression**		
Vaccinated	1.72 (0.44–6.96)	0.458
Not vaccinated	Reference	Reference
**Cognitive deficit**		
Vaccinated	0.15 (0.03–0.87)	0.034*
Not vaccinated	Reference	Reference
**Tics**		
Vaccinated	0.93 (0.19–4.57)	0.925
Not vaccinated	Reference	Reference

Response	B (95% CI)	*p value*
**Quality of Life (EQ-5D-5L Assessment)**		
Vaccinated	− 2.11 (− 4.21 to − 0.20)	0.034*
Not vaccinated	Reference	Reference
**General Health Status (EQ-VAS)**		
Vaccinated	6.00 (− 4.00 to 16.00)	0.236
Not vaccinated	Reference	Reference
**MMRC Scale**		
Vaccinated	− 0.19 (− 0.86 to 0.47)	0.565
Not vaccinated	Reference	Reference
**BCSS**		
Vaccinated	0.00 (− 1.18 to 1.15)	0.982
Not vaccinated	Reference	Reference

This table describes the differences in PACS occurrence among COVID-19 survivors between vaccinated and nonvaccinated patients.

P value < 0.05.

*PTSD* Posttraumatic stress disorder, *VAS* Visual Analogue Scale, *EQ-5D-5L* EuroQol 5-dimension 5-level assessment, *MMRC* Modified Medical Research Council scale, *BCSS* Breathlessness, Cough, and Sputum Scale.

## Discussion

To our knowledge, this is the first cohort study that evaluated PACS symptoms according to the time of COVID-19 infection. This study is also the first to evaluate tics as one of the components of PACS and the effect of COVID-19 vaccination on PACS symptoms.

According to our results, a higher prevalence of PTSD, anxiety, depression, cognitive deficit and tics was reported among COVID-19 survivors than among non-COVID-19 controls. COVID-19 survivors had a prevalence of PTSD, anxiety and depression of 84%, 38.4% and 75.2%, respectively, with a lower prevalence noted among non-COVID-19 controls of 59.7%, 19.3% and 45.5%, respectively. Similarly, studies reported that after COVID-19 infection, patients experienced high levels of anxiety, depression, and PTSD, with a prevalence of approximately 22%^27^. Although our study showed higher levels of PTSD, anxiety and depression among COVID-19 survivors, we also reported notable levels of the aforementioned conditions among non-COVID-19 controls, indicating that the pandemic had an impact on the general population regardless of their history of COVID-19 infection. This can be explained by the considerable mental health sequelae of the COVID-19 pandemic restrictions on COVID-19 patients as well as on people who were never infected^28^. In concordance with our findings, studies showed that cognitive deficit, including memory loss and concentration difficulties, was experienced by COVID-19 survivors^29^. These cognitive symptoms were associated with the presence of hypometabolic clusters in the brains of patients with PACS, as demonstrated using fluorodeoxyglucose (FDG)-positron emission tomography (PET)^30^. In addition, a recent case report described a patient that presented with a movement disorder after COVID-19 infection^14^. Previous studies showed a surge in tics and tic-like attacks during the COVID-19 pandemic, suggesting that this surge was due to the stress induced by the pandemic^16^. However, our study is the first to show that these tic-like attacks occurred at a higher frequency among COVID-19 survivors. Moreover, our results revealed that COVID-19 survivors had significantly lower EQ-5D-5L assessment and EQ-VAS scores, which indicates a lower quality of life and general health status. Additionally, our study revealed that COVID-19 survivors had higher BCSS scores, indicating a higher incidence of respiratory symptoms. However, mMRC scale scores were not significantly higher among COVID-19 survivors. Since the BCSS evaluates respiratory symptoms in a more comprehensive way than the mMRC scale by including sputum and cough, our results indicate that respiratory symptoms of PACS are not only driven by dyspnea but also by cough and sputum and are hence better assessed by the BCSS. Similar to our results, Zhang et al. illustrated that respiratory symptoms are common among COVID-19 survivors^31^.

Our analysis models demonstrated that COVID-19 survivors experienced a higher prevalence of depression, cognitive deficit, tics and respiratory symptoms during the first 6 months after the onset of infection. Likewise, impaired general health status was also prevalent during the first 6 months after infection. On the other hand, impaired quality of life, PTSD and anxiety were not significantly more prevalent among COVID-19 survivors until more than 6 months had elapsed from the onset of the infection. All these findings except for respiratory symptoms were prevalent 6–12 months and 12 months after the onset of infection. Comparable to our findings, studies showed that PACS symptoms persisted for up to 12 months after the onset of infection^32^. Additionally, it was shown that in more than 90% of patients, the time to recover from symptoms exceeded 9 months from the onset of infection^33^. Furthermore, a study on COVID-19 support groups demonstrated that after 6 months of follow-up, PACS symptoms persisted in a large proportion of patients^34^. However, it is important to mention that in our study, the peak in these symptoms (highest OR/B) was in the 6–12 month period, indicating that these symptoms increased from the onset of infection until 12 months, after which the prevalence decreased. The lower prevalence of PACS symptoms 12 months after the infection could be attributed to the fact that with time, the immune dysregulation status and the high cytokine levels described among PACS patients start to resolve, which was also described by Huang et al.^31^. Similarly, a study showed that the prevalence of PACS among the study patients increased from 27.8% at 4 months after the infection to 34.8% at 7 months^34^. Other studies showed that approximately half of the patients with PACS did not have any symptoms in the first 3 months after infection^12^. The neuropsychiatric symptoms were prevalent for much longer than respiratory symptoms, which can be explained by several hypotheses. One of these includes the bidirectional overlapping relationship between cognitive and psychiatric symptoms^35^, as it is well known that psychiatric symptoms, including stress, anxiety and depression, which persist due to the continued social and economic sequelae of the pandemic, might affect cognitive function^36,37^. Additionally, the pathophysiology of neuropsychiatric symptoms was attributed to the ability of the virus to penetrate and infect brain cells^35^. However, the relationship between the immune system and the brain is different from other organs in the body, as the brain has fewer immune cells and lymphatic systems, which might result in a delay in completely clearing the infection compared to organs highly enriched with lymphatics, such as the respiratory system^38^. Furthermore, to the best of our knowledge, this is the first study to report a reduction in symptoms 12 months after the onset of infection. Our results suggest that the absence of respiratory symptoms during clinical evaluation does not rule out the possibility of the development of neuropsychiatric symptoms. Additionally, the changes in symptom prevalence according to the time of infection demonstrated by our study might impact how clinicians address these symptoms. If PACS symptoms during the first 6 months after the onset of infection are absent, the patient must be screened for these symptoms 6–12 months after the infection, as the highest prevalence of these symptoms occurs in the period between 6 and 12 months after infection.

Although the pathogenesis of PACS is still not well understood, several hypotheses explaining this syndrome have been generated by observational studies^39^. Several studies showed that postvaccination breakthrough infections were associated with a reduction in the risk of the development of PACS symptoms^40–42^. However, our study is the first to report a reduction in the cognitive symptoms of PACS and quality of life impairment after COVID-19 vaccination. Symptoms other than those associated with cognitive function and quality of life impairment did not improve after vaccination. This finding can be explained by the fact that cognitive deficit was the only factor evaluated using an objective test (MoCA), as other symptoms were assessed using subjective tools. The reduction in PACS symptoms after COVID-19 vaccination can be explained by the formation of antibodies that can eliminate the persistent SARS-CoV-2 infection that was detected in a large number of PACS patients^15^.

This study had several strengths. Despite the fact that several studies have been conducted to explore PACS, few studies have focused on nonhospitalized patients. In addition, this is one of few studies to evaluate the changes in PACS symptom prevalence with time and to assess the newly reported symptoms of PACS, i.e., tics. Additionally, the use of comprehensive tools that were shown to be reliable in assessing PACS symptoms by Johns Hopkins Hospital is another strength. However, our study had several limitations. First, the moderate response rate, which resulted in a small sample size, could have introduced bias to the results of this study. This low response rate can be explained by the long interview that had to be conducted and the stigma surrounding mental health, as several of our outcomes were related to mental health. In addition, the lower number of COVID-19 survivors could be because the percentage of patients who were infected with COVID-19 comprises only 16% of the Jordanian population. Second, this was a single-center study, which limits the generalizability of our findings. Third, we did not have the baseline status of our outcome measures for COVID-19 survivors. Fourth, the retrospective design of this study can only predict association but not causality between the variables of the study. However, the retrospective design by its nature allows for long follow-up after the onset of COVID-19 infection. Although our models were adjusted for several confounders, the risk of confounding bias cannot be fully excluded. Furthermore, the ascertainment of COVID-19 survivors and noninfected cohorts was based on RT‒PCR testing. Since not all patients within the control group underwent RT‒PCR testing or serological tests, it was possible that some patients who were included in our noninfected cohort were previously infected with COVID-19, especially those with an asymptomatic infection who were less likely to be tested. However, the COVID-19 surveillance programs in Jordan included daily random testing, including asymptomatic patients, which might reduce the risk of ascertainment bias in our study. Including patients admitted to the hospital ward during the interview, which was after COVID-19 infection recovery, along with outpatients in our cohort is another limitation, as hospitalization during the interview time might result in several symptoms similar to PACS symptoms, and a subgroup analysis according to hospitalization status during the interview was not possible due to the small sample size. The reason behind including hospitalized patients was because they are more likely to tolerate long interviews compared to visitors of outpatient clinics. However, our results showed that there was no significant difference in the patients who were admitted to the hospital ward during the interview between the COVID-19 and control cohorts; hence, this is less likely to affect our results. Finally, we did not have any data about COVID-19 severity among COVID-19 survivors.

In conclusion, more than 1 year after the onset of COVID-19 infection, nonhospitalized COVID-19 survivors are still suffering from a higher prevalence of PTSD, anxiety, depression, cognitive deficit, and tics as well as lower quality of life and general health status than non-COVID-19 controls. However, it is important to mention that most of the symptoms reached their highest prevalence in the 6–12 month period and declined after the 12 month period. In addition, the respiratory symptoms were not different between the two study groups 6 months after the infection. Moreover, COVID-19 vaccination was shown to significantly decrease the prevalence of cognitive deficit and quality of life impairment among COVID-19 survivors. Longitudinal studies with long follow-up periods are needed to establish the time that should elapse after COVID-19 infection for the symptoms of PACS to subside and to better characterize the nature of PACS with a focus on tics. Additionally, randomized controlled clinical trials are needed to assess the possibility that COVID-19 vaccines might relieve PACS symptoms.

## Supplementary Information

Supplementary Information 1.

Supplementary Information 2.

## Data Availability

The data related to this article are available from the corresponding author upon reasonable request.
