# Supplementary material for: New symptoms and prevalence of postacute COVID-19 syndrome among nonhospitalized COVID-19 survivors
Source: Sci Rep. 2022 Oct 8;12:16921. doi: 10.1038/s41598-022-21289-y (PMC9547088; doi:10.1038/s41598-022-21289-y)
Supplement: Supplementary file 2 — Supplementary Information 2. [file 41598_2022_21289_MOESM2_ESM.doc]

**Participants Flow Diagram**

**Asertainment**

**Analysis**

**Enrollment**

Assessed for eligibility (n=730)

Excluded (n=258)

♦  Declined to participate (n= 200)

♦  Withdrew consent during the interview (n= 22)

♦  Did not meet the inclusion criteria (n=36)

Control (n=347)

Control (n= 347)

COVID-19 Survivors (n=125)

COVID-19 Survivors (n= 125)

Included (n=472)
